# 

**Published:** 1893-01

**Authors:** 


### TABLE OF CONTENTS.

| | PAGE. |
|---|---|
| A "Bed-day" for Tired Ones | 207 |
| About the Teeth | 205 |
| Abuse of Cocaine | 66 |
| Advancement of Women, The | 73 |
| A Life Saving Thought | 131 |
| Age of the Earth, The | 42 |
| Against Pungent Odors | 161 |
| Animal Food | 212 |
| Are Animals Immortal ? | 30 |
| Avoidable Diseases | 225 |
| Animal and Invalid Diet | 235 |
| Behind the Counter | 7 |
| Behind the Counter | 32 |
| Behind the Counter | 51 |
| Behind the Counter | 78 |
| Behind the Counter | 114 |
| Behind the Counter | 132 |
| Bread and Dyspepsia | 184 |
| Beef Tea | 40 |
| Care of the Hands | 14 |
| Care of the Baby | 89 |
| Care of the Eye-brows | 211 |
| Chemistry of the Table | 202 |
| Chapter on Wrinkles, A | 80 |
| Coolings | 104 |
| Cold Bathing | 137 |
| Correct Sitting | 211 |
| Crimean Ghost Story, A | 15 |
| Cross-Thinkers | 28 |
| Cold Hands and Feet | 235 |
| Dangers of Eating Fresh Bread | 238 |
| Death by Violence | 17 |
| Diphtheria | 57 |
| Docking Horses Tails | 88 |
| Don't Sleep in Linen | 208 |
| Draughts and Deaths | 134 |
| Dust | 38 |
| Dyspepsia | 257 |
| Editorial, "Hub Me" | 268 |
| Editor's Outlook | 243 |
| Editor's Outlook | 118 |
| Editor's Outlook | 140 |
| Editor's Outlook | 162 |
| Editor's Outlook | 189 |
| Editor's Outlook | 219 |
| Effects of Environment | 139 |
| Epilepsy | 148 |
| Eyes, The | 14 |
| Family Order | 154 |
| For Tender Feet | 213 |
| For the Complexion | 140 |
| Fragment from Carlyle, A | 44 |
| Fruit Diet | 207 |
| Free or Forced Vaccination | 58 |
| Future Life, The | 90 |
| General Notes | 269 |
| Germ Diseases | 17 |
| Gray Hairs | 12 |
| Gruel | 40 |
| Glass of Water at Bed-time, A | 237 |
| Health and Civilization | 232 |
| Health Resorts and Mineral Waters | 174 |
| Healthy Bed-clothing | 64 |
| Hiccough | 187 |
| House and Home | 238 |
| House and Home | 214 |
| House and Home | 262 |
| How to Make Eye-water | 213 |
| Hot Milk | 212 |
| How to Restore Drowning Persons | 37 |
| Hot Milk as a Stimulant | 138 |
| How to Improvise a Vapor Bath | 89 |
| How to Fumigate a Room | 188 |
| How She Keeps Well | 208 |
| How to Make an Invalid's Bed | 211 |
| Hygiene Hints for Railroad Travel | 193 |
| Hygiene of Old Age | 25 |
| Hurried Dinners | 87 |
| Hysteria in Children | 236 |
| Infectiousness of Tuberculosis | 13 |
| Infectious Diseases | 82 |
| Infectious Diseases | 136 |
| Infection from Soil | 187 |
| In the Sick Room | 218 |
| Keeping Fruit and Vegetables | 88 |

PAGE.
Leave-taking .... .. ....... .... ..242
Literary.... ....................... 22
Literary................. ....... 47
Literary.... .. .... ............ 71
Literary........................ 96
Literary..... ....................119
Literary.........................142
Literary.... ..... ...............164
Literary................ .. ......190
Literary.........................220
Literary........................ 243
Literary....... ......... .... .. ..269
Liberal Use of Butter .............183
Lime Juice Better than Vinegar.... .188

Manners of Children ............... 43
Meat Harmful in Summer...........139
Medicine Taking..................156
Medical Etiquette... ...............117
Mental Dyspepsia... ...............200
Mesmerism Extraordinary.......... 41
Milk Instead of Medicine...........160
Milk as a Food................ ....209
Miscellaneous................. .. 20
Miscellaneous ..................... 45
Miscellaneous..................... 68
Miscellaneous........ .......... 92
Miscellaneous. ..................259
Mound Builders....... .......... 53

Nervous System, The.............228
New Year, The..... .. .......... 1
Night Air.........................159

Onions... ....... .......,......113
Opium Curse, The................ 9
Oranges as a Medicine...... .....158
Over-eating........................185
Over-work and Under-work .........206

Pains and their Significance..... ...209
Perils of Investigation............. 5
Perspiration................ ....189
Physical Culture and Its Abuse in Foot-Ball........ .. ..............251
Poisonous Milk............ ......102
Popular Errors in Medicine...... ....145
Popular Fallacies..................121
Prevention of Juvenile Smoking... .. 18
Proper Care of Infants.......... ...110
Preservation of Beauty..............179
Physical Perfection................ 11
Physical Necessities..... ......... 49
Pulpit Defence of the Stage......... 65
Physiological Basis of Labor ........128

PAGE.
Rapid Motion of Food.............. 11
Recreation Bureau Notes............143
Recreation Bureau Notes............166
Rest and Disease....... ......... 8
Remarkable Case of Fingers Re-uniting.... .. ..................... 86
Rural Life.. ...................... 33

Saved His Friends and Died........ 63
Sea Bathing............... ......186
Shell Fish for Invalids...... ......138
Sleep ............................ 19
Sleep... ......................... 112
Some Aspects of Suicide............169
Smoking by Boys.... .............. 61
Sneezes and Stitches........... ... .113
Sultan's Food, The................ 19
Spring Diseases...................109
Stranger than Fiction............... 67
Summer Clothing...................140
Surfeit, A.......... ........ ....112
Superstitious Remedies............. 91
Squinting......................... 36
Should the Skins of Fruit be Eaten?..237

The Laws of Health..... .. ......213
The Indulgence of Grief............182
Teplitz Mineral Water......... ....196
Tannin in Tea ..................... 64
The Fatal Camel................... 90
The Living and the Dead...........106
The Difference.....................113
To Boys who Smoke............... 37
Two Cases of Hypnotic Suggestions..116
To Cure a Cough..................210
Town and Country.... ........... 55
Town and Country ................ 84
Tobacco ............... ...... 75
Theory of Human Action........ ..184

Uncomfortable People.............. 62

Warts......................... ..113
Waste of Force.... .. ............187
Water as a Medicine............... 39
Wet Feet and Colds............... 213
Women in the Sick Room........... 59
When to Eat....................... 3
When to Marry... ................. 35
What is Cholera ?....... ........ 97
What is Cholera ?.................124
What is Cholera ?.... .. ..........150
When Things Get in the Eye....... 209
Whooping Cough........ ........138
Wrinkles............ ........ ...186

### LITERARY.

HANDBOOK OF EMERGENCIES AND COMMON AILMENTS. By E. F. Bradford, A. B., M. D., assisted by Louis Lewis, M. D. B. B. Russell, Boston, Publisher, 4to 450 pp. Sold by subscription only.

This is one of the most valuable of popular medical works, which has come under our notice, not because of the subjects treated of, so much as the clear and easily understood manner of presenting them to the unprofessional reader, thus qualifying him to treat with requisite skill a subject whose sudden injuries or attack require the immediate application of remedies. The illustrations, which are many, show the best mode of adjusting splints and bandages to simple and compound fractures, checking the flow of blood from arterial wounds, hemorrhages, lacerated or contused wounds, etc. We commend the work also for its great simplicity and thoroughness, rendering it of equal value to the skillful and the unskillful in emergencies which are likely to occur at most unexpected moments.

AL-MODAD, OR LIFE SCENES BEYOND THE POLAR CIRCUMFLEX. By an Untrammelled Free-Thinker. M. Louise Moore and M. Beauchamp, Publishers, Cameron Parish, La.

This book is claimed to be "A Religio-Scientific solution of the problems of present and future life." Perhaps it is.

PHTHISIS; A NEW METHOD OF TREATMENT. By Henry S. Norris, M. D., N. Y.

Consumption, among diseases, is the one most dreaded. It has for the most part been held incurable. By a statistical summary taken from the consus of 1890 there were in a single year, 101,645 deaths from this cause alone. This pamphlet recommends the internal application of Ozone as a remedy, and cites many instances of relief growing out of its use.

TRANSACTIONS OF THE AMERICAN OTOLOGICAL SOCIETY. 25th Annual meeting, at New London, Conn.

To physicians in active practice this volume of some 400 pages will be found extremely useful and reflects great credit upon the Society for its thoroughness of statement of ear afflictions and cures.

THE POWER OF THOUGHT IN THE PRODUCTION AND CURE OF DISEASE. By Wm. H. Holcombe, M. D., Chicago. Purdy Pub. Co., Chicago, 21 pp. Price, 15 cents.

This is a timely treatise, valuable for its truth, which ought to be better understood by the practical healers of disease.

THE CHOICE BETWEEN EXTIRPATION AND COLOTOMY IN COURSE. By Charles B. Kelsey, M. D., New York.

This is a pamphlet of 8 pp., conveying much valuable information upon a subject of great importance to surgeons.

NOMENCLATURE OF CERTAIN EAR AND NASAL DISEASES.

This catalogue is a little too much for us, but shows how wonderfully and fearfully we are made, to require such a string of latinized terms to describe the complex ailments of two rather necessary organs.

THE WORLD ALMANAC, which we offer as a premium, will be issued about the 20th of January. It will contain mention of all the leading events of the past year the world over, with a vast deal of other important information. Its index comprises more than 1000 subjects. No one having this book can plead ignorance of what is transpiring any where within reach of the telegraph or the telescope. Send in your orders. Hall's Journal was never so prosperous as now.

---

ABSOLUTELY PURE.—This is a rare quality in a BAKING POWDER, but the ROYAL claims it, and so far as we know, the claim is undisputed; at all events it has won the favor of pastry cooks and bakers the world over, and keeps its place as the acknowledged leader of the clan.

---

### THE DAILY CALENDAR

Of the Pope Mf'g Co., of Boston, for 1893, deserves mention as a practical business calendar for the year. For eight consecutive years, this company has issued what is known as the Columbia Desk Calendar and Stand, consisting of a pad of 366 leaves, one for each day in the year, and one for the entire year. The pad rests upon a metallic stand, arranged to take up very little desk room. Send in your orders and learn all about bicycles.

---

### THE CENTURY.

What would you do if you were a stranger in London, with no money except a million-pound bank note in your pocket, and some good reasons why you were afraid to go to the Bank of England and get it changed? This is the theme of Mark Twain's story, "The £1,000,000 Bank Note," which will be printed in the January *Century*.

---

### A PURE COCOA.

The Breakfast Cocoa prepared by Messrs. Walter Baker & Co., Dorchester, is generally recommended by physicians as the best drink for the morning meal. The manufacturers warrant this cocoa to be absolutely pure and soluble, the excess of oil being properly and carefully removed. It is guaranteed to have three times the strength of ordinary cocoa, because the latter is invariably mixed with sugar or arrowroot. It costs less than one cent a cup. It is delicious, nourishing, strengthening, and easily digested. For sale by all grocers in the United States.

---

### FOOD.

This elegant monthly household magazine has just published "A Yard of Sweet Clover," a frieze 5¾ x 36 inches long, representing a gracefully blended collection of clover blossoms, as a holiday offering to its subscribers.

---

### A NEW ENTERPRISE.

The Press Claims Company, whose advertisement appears in another column will become familiar to our readers during the coming year. It illustrates the advantages of co-operation on an extensive scale, being a combination of hundreds of the leading newspapers of the United States, for the purpose of protecting their patrons against unscrupulous claim agents, and securing prompt, efficient, and economical service to all persons having dealings with the Government. It will secure patents and pensions, perfect land titles and attend to all other legitimate business of the kind on terms that will make its employment highly advantageous.

Membership in this company is a guarantee of the high standing of any newspaper, all applications being carefully considered and passed upon by the Board of Directors before allotment. The company comes before the public backed by the collective endorsement of over five hundred of the leading journals of the United States. That this journal has been admitted to the association is an advantage of which such of our readers as have transactions with the Government will be glad to avail themselves of.

